# Intraoperative neuromonitoring and other techniques limiting the number of complications in thyroid surgery

**DOI:** 10.1186/1756-6614-8-S1-A7

**Published:** 2015-06-22

**Authors:** Marek Dedecjus

**Affiliations:** 1 Department of Oncological Endocrinology and Nuclear Medicine, Center of Oncology, Maria Skłodowska-Curie Memorial Institute, Warsaw, Poland

## 

Evolution of surgical techniques, together with progress in other fields of medicine (particularly in anesthesiology) limited - practically to zero - the mortality in thyroid surgery. Further development in thyroid surgery is directed towards limitations of postoperative complication rates.

The specific complications after thyroid surgery are hypoparathyroidism and recurrent laryngeal nerve (RLN) palsy (uni- or bilateral). The former, although mostly transient in character, has a frequency reaching up to 60% in cases of total thyroidectomy with central neck dissection because of thyroid cancer. At present, only meticulous preparation by experienced surgeons, together with PTH and calcium concentration monitoring, may be helpful in prevention and early diagnosis of hypoparathyroidism. Subsequent substitutive therapy with calcium and vitamin D metabolites should prevent hypocalcaemia.

Recent two decades was the time of intensive development of intraoperative neuromonitoring (IONM) of laryngeal nerves. Nowadays this technique, although still developing, has a stable place in thyroid surgery. Intraoperative neuromonitoring - at its present form - cannot prevent laryngeal nerve palsy, nevertheless together with stage thyroidectomy and careful preparation allows to avoid the most serious complication in thyroid surgery - bilateral RLN palsy. Recently developed continuous intraoperative neurmonitoring of vagal nerve is a promising tool and probably the next step in modern prevention of RLN palsy. Efforts in further development and standardization of the technique will hopefully result in the technique of IONM, allowing not only to intraoperatively diagnose but also to prevent RLN palsy. Further efforts are needed in limiting the invasiveness of the procedure.

